# Comparative analysis of diguanylate cyclase and phosphodiesterase genes in *Klebsiella pneumoniae*

**DOI:** 10.1186/1471-2180-12-139

**Published:** 2012-07-09

**Authors:** Diana P Cruz, Mónica G Huertas, Marcela Lozano, Lina Zárate, María Mercedes Zambrano

**Affiliations:** 1 Molecular Genetics, Corporación Corpogen, Carrera 5 No. 66A-34, Bogotá, Colombia; 2 Department of Biological Sciences, Universidad de los Andes, Bogotá, Colombia; 3 Department of Biological Sciences, Pontificia Universidad Javeriana, Bogotá, Colombia

**Keywords:** *Klebsiella pneumoniae*, Biofilm, Diguanylate cyclase, Phosphodiesterase, c-di-GMP

## Abstract

**Background:**

*Klebsiella pneumoniae* can be found in environmental habitats as well as in hospital settings where it is commonly associated with nosocomial infections. One of the factors that contribute to virulence is its capacity to form biofilms on diverse biotic and abiotic surfaces. The second messenger Bis-(3’-5’)-cyclic dimeric GMP (c-di-GMP) is a ubiquitous signal in bacteria that controls biofilm formation as well as several other cellular processes. The cellular levels of this messenger are controlled by c-di-GMP synthesis and degradation catalyzed by diguanylate cyclase (DGC) and phophodiesterase (PDE) enzymes, respectively. Many bacteria contain multiple copies of these proteins with diverse organizational structure that highlight the complex regulatory mechanisms of this signaling network. This work was undertaken to identify DGCs and PDEs and analyze the domain structure of these proteins in *K. pneumoniae*.

**Results:**

A search for conserved GGDEF and EAL domains in three sequenced *K. pneumoniae* genomes showed that there were multiple copies of GGDEF and EAL containing proteins. Both single domain and hybrid GGDEF proteins were identified: 21 in *K. pneumoniae* Kp342, 18 in *K. pneumoniae* MGH 78578 and 17 in *K. pneumoniae* NTUH-K2044. The majority had only the GGDEF domain, most with the GGEEF motif, and hybrid proteins containing both GGDEF and EAL domains were also found. The I site for allosteric control was identified only in single GGDEF domain proteins and not in hybrid proteins. EAL-only proteins, containing either intact or degenerate domains, were also identified: 15 in Kp342, 15 in MGH 78578 and 10 in NTUH-K2044. Several input sensory domains and transmembrane segments were identified, which together indicate complex regulatory circuits that in many cases can be membrane associated.

**Conclusions:**

The comparative analysis of proteins containing GGDEF/EAL domains in *K. pneumoniae* showed that most copies were shared among the three strains and that some were unique to a particular strain. The multiplicity of these proteins and the diversity of structural characteristics suggest that the c-di-GMP network in this enteric bacterium is highly complex and reflects the importance of having diverse mechanisms to control cellular processes in environments as diverse as soils or plants and clinical settings.

## Background

*Klebsiella pneumoniae,* an opportunistic pathogen responsible for a wide range of nosocomial infections that include pneumonia, bacteremia and urinary tract infections, is estimated to cause approximately 8% of hospital acquired infections [1-5]. This Gram-negative bacterium can also be found in the environment in association with plants, as well as in soil and in water [2,6]. One important factor associated with virulence in *K. pneumoniae* is its capacity to adhere to surfaces and form biofilms. Although the formation of biofilms by *K. pneumoniae* is still not fully understood, several key determinants have been identified such as pili, polysaccharides, quorum sensing and transport and regulatory proteins [7-13]. More recently, it has been shown that c-di-GMP controls type 3 fimbria expression and biofilm formation in *K. pneumoniae* by binding to and modulating the activity of the transcriptional regulator MrkH [14,15]. The second messenger c-di-GMP is known to play a key role in several cellular functions as well as in biofilm formation in bacteria where it modulates the transition between planktonic and sessile lifestyles. Low levels of c-di-GMP result in increased motility while high levels promote adhesion to surfaces, production of exopolysaccharides and biofilm formation [16,17].

The intracellular levels of c-di-GMP are regulated by the antagonistic activity of diguanylate cyclase (DGC) enzymes and phosphodiesterases (PDEs) that catalyze synthesis and hydrolysis of this molecule, respectively [16,18]. Several genetic and biochemical studies have shown that besides their C-terminal catalytically active A site, most of these proteins harbor N-terminal sensory domains that can respond to different internal and external signals, triggering activation of DGCs or PDEs. When enough c-di-GMP is available, it binds different effector molecules, proteins or RNAs, which influence cell behavior [18]. The active site of DGCs contains a conserved GGDEF domain, characterized by the GG(D/E)EF motif, while PDE activity is associated with C-terminal EAL or HD-GYP domains [16,17]. These domains can be found separately or together, forming hybrid proteins that have both GGDEF and EAL domains. Hybrid proteins usually have either PDE or DGC activity, although in some cases both functions are apparently present [17,18]. DGCs can also be subject to allosteric product inhibition by c-di-GMP, which binds to a secondary site (I site) separated from the A site by 5 amino acids [16]. This feedback control helps to maintain adequate pools of c-di-GMP, avoiding excessive consumption of the GTP substrate and reducing stochastic perturbations in cellular c-di-GMP content [16,17]. GGDEF and EAL proteins can also contain one or more transmembrane regions and signal peptides that can anchor these proteins to the membrane, most probably allowing physical isolation of different GGDEF and EAL systems to unique microenvironments [17]. In addition, some bacterial species can harbor multiple copies of proteins with GGDEF and EAL domains. Many of these copies may contain degenerate sites that are inactive and do not directly synthesize or degrade c-di-GMP but have adopted alternative functions, either as c-di-GMP binding effector proteins or through direct macromolecular interactions with no involvement of c-di-GMP at all [17]. The diversity of sensor domains coupled to the multiplicity of these genes reveal a complex c-di-GMP network that integrates diverse environmental and cellular signals [16,17].

This work was carried out to identify GGDEF and EAL domain-containing genes in three sequenced *K. pneumoniae* genomes. Searches were done for the conserved GGDEF/EAL domains and the RxxD allosteric I site. Sensory domains associated with these proteins, as well as transmembrane helices and signal peptides were also identified. The results show that there are multiple copies of these genes in the sequenced genomes studied and that some of these are shared while others are unique to a particular strain.

## Results and discussion

### Multiplicity of genes encoding GGDEF and EAL containing proteins

**Figure 1:**
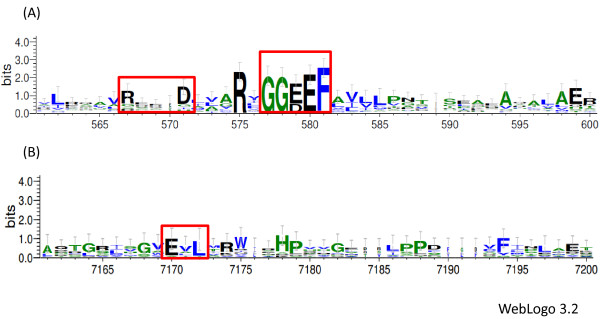
**Logo sequences for DCG and PDE domains.** Logos are shown for the active DGC domain and the I site (**A**) and the PDE domain (**B**). Red rectangles show the conserved A site (GGEEF or GGDEF), the I site (RxxD), and the EAL domain. The error bars indicate an approximate, Bayesian 95% confidence interval.

To have an inventory of the number of genes coding for GGDEF and EAL domain-containing proteins, PSI-BLAST was used to identify the conserved GG(D/E)EF and E(A/V)L motifs in the three sequenced *K. pneumoniae* genomes. The genomes available at the time this analysis was done included one environmental strain, *K. pneumoniae* Kp342, a nitrogen-fixing endophyte isolated from corn [6], and two clinical isolates from the same subspecies: *K. pneumoniae* subsp. *pneumoniae* MGH 78578, isolated from a patient with nosocomial pneumonia [6], and *K. pneumoniae* subsp. *pneumoniae* NTUH-K2044, isolated from a patient with a hepatic abscess and meningitis [19]. All genomes had multiple copies for proteins with GGDEF domains: 17 for NTUH-K2044, 18 for MGH 78578 and 21 for the environmental isolate Kp342 (Table 1). The majority of these proteins contained the GGEEF sequence motif and only 30% had GGDEF (Figure 1). A subset of the proteins (29%) had both GGDEF and EAL domains and more than 50% of these had GGDEF degenerate domains. Two GGDEF-only proteins (KPK_A0039 and KPN_pKPN3p05901) had GGDEF degenerate domains and were found on plasmids. Multiple copies of proteins with single EAL domains were also identified: 15 for the environmental isolate Kp342, 15 for MGH 78578 and 10 for NTUH-K2044 (Table 1). Most of these proteins (61%) had an intact EAL domain, including the EVL motif (Figure 1), and 39% had EAL degenerate domains (Table 1). Some of the EAL degenerate proteins, such as KPK_A0040 and KPN_pKPN3p05966, were found on plasmids.

**Table 1:** **List of domains found in the genomes of*K. pneumoniae*342, MGH 78578 and NTUH-K2044**

	***K. pneumoniae*342**			***K. pneumoniae*MGH 78578**			***K. pneumoniae*NTUH-K2044**			**Total Predicted DGC**	**Total Predicted PDE**
	**GGDEF**	**GGDEF + EAL**	**EAL**	**GGDEF**	**GGDEF + EAL**	**EAL**	**GGDEF**	**GGDEF + EAL**	**EAL**		
Total proteins	15	6	15	13	5	15	12	5	10	56	56
Transmembrane segments	11	5	6	9	4	7	8	4	6	41(73%)	32 (57%)
Signal peptides	1	1	1	1	0	1	0	0	1	3 (5%)	4 (7%)
With GAF domain	3	0	1	4	0	0	3	0	0	10 (18%)	1 (2%)
With HAMP domain	2	1	0	2	1	0	1	1	0	8 (14%)	3 (5%)
With PAS domain	1	1	0	1	1	0	1	1	0	6 (11%)	3 (5%)
With BLUF domain	0	0	3	0	0	2	0	0	2	0	7 (12%)
With MASE domain	2	1	1	0	1	1	0	1	1	5 (9%)	6 (11%)
With CACHE domain	1	0	0	2	0	0	2	0	0	5 (9%)	0
With CHASE domain	0	1	0	0	0	0	0	0	0	1 (2%)	1 (2%)
With CSS-motif domain	0	0	5	0	0	6	0	0	5	0	16 (28%)
With sensor domains	9	4	10	9	3	9	7	3	8	35 (62%)	37 (66%)
With allosteric I site	7	0	0	7	0	0	5	0	0	19 (34%)	0
With degenerate GGDEF	1	3	0	1	3	0	0	3	0	11 (20%)	9 (56%)*
With degenerate EAL	0	2	6	0	2	6	0	2	4	6 (38%)*	22 (39%)

*Average calculated based only on the number of hybrid proteins (16).

Numbers in parenthesis indicate % of total for either DGCs or PDEs.

To further characterize these proteins, signal peptides, sensor and conserved domains were identified. Only 5% of GGDEF and 7% of EAL proteins in *K. pneumoniae* included signal peptides (Table 1), indicating that they could be transported across or anchored in membranes [20,21]. A larger proportion of the proteins contained transmembrane segments, 73% of the GDDEF and 57% of EAL-containing proteins (Table 1), suggesting that regulation and/or enzyme activity is most likely occurring at the membrane, as has been suggested [22,23].

### Sensor domains found in GGDEF and EAL containing proteins

One of the most intriguing aspects of the enzymes involved in modulating intracellular levels of c-di-GMP is their modular structure characterized by the presence of additional input sensory domains [24]. Therefore, a search was carried out for the diverse periplasmic, cytoplasmic, and integral membrane domains that have been described [23,25]. Most of the GGDEF and EAL-containing proteins in *K. pneumoniae* contained sensor domains, 62% and 66%, respectively (Table 1). Some domains were found exclusively or predominantly in GGDEF proteins (CACHE, PAS and GAF) or EAL proteins (BLUF and CSS), while others were shared or found in hybrid proteins (HAMP, CHASE and MASE) [Additional file 1. As in other bacteria, the different sensor domains suggest a diverse range of environmental stimuli involved in regulatory responses in this bacterium [26,27] (Table 1). In GGDEF proteins the most frequently found domain was GAF (18%) (cGMP phosphodiesterase, adenylyl cyclase), a cytoplasmic sensor domain that can bind a number of small molecules including monocyclic nucleotides and oxygen and that is also common in signal transducing photoreceptor proteins such as phytochromes, which covalently link chromophores [28]. This was followed by HAMP (Histidine kinases, Adenylyl cyclases, Methyl binding proteins, Phosphatases) domain-containing proteins (14%). This domain has been found in many transmembrane receptors where it transmits signals from periplasmic sensor domains to cytoplasmic output domains via conformational changes [25,29]. The PAS (PER, *A*RNT and SIM) domain was found only in 11% of the GGDEF proteins. PAS is structurally similar to GAF and can bind small molecules such as heme, flavin, and adenine [29,30]. Other domains were also found in smaller proportions. The membrane-embedded MASE (Membrane-associated sensor) domain [25] was identified in 9% of the GGDEF proteins and 11% of the EAL proteins (Table 1), and the extracellular CHASE (cyclase/histidine kinases-associated sensing extracellular) and CACHE (Ca2+ channels and chemotaxis receptors) domains were found in 2% and 9% of the cases, respectively. The CHASE domain apparently recognizes short peptides and cytokines [25,30,31]. The CACHE domain is involved in binding small ligands such as amino acids, sugars and organic acids, and has been found in prokaryotic chemotaxis receptors and animal ion channels [30,31]. The most common sensor domain in EAL proteins was the CSS-motif (28%) of unknown function, followed by BLUF (for ‘sensing blue-light using FAD’) (12%), which is involved in sensing blue-light and possibly redox states [32]. Some sensor domains identified in other bacteria were not found in *K. pneumoniae*, as was the case for REC (receiving domain with phosphoacceptor site), which is implicated in activation of DGC proteins in organisms such as *Caulobacter crescentus* and *Pseudomonas*[27].

### Predicted catalytic activity in GGDEF-containing proteins

Active DGCs consist of two subunits, each with an A site that binds a GTP molecule at the interface between the two subunits. The A site has the characteristic conserved GGDEF or GGEEF motif and point mutations that affect this sequence abolish enzymatic activity [17]. Many DGCs are also subject to allosteric inhibition, which involves binding of c-di-GMP to the I site characterized by the RxxD motif [16,17]. Mutations of the R residue alter the inhibitory function and allosteric control, while mutations of the D amino acid do not [16]. In *K. pneumoniae* 80% of the identified GGDEF-containing proteins had an intact conserved A site (Figure 1) and of these, only 34% had the conserved I site motif (RxxD) (Figure 1, Table 1), which was present only in single-domain GGDEF proteins. Interestingly, the majority of the proteins that lacked the I site had the GGDEF sequence, which is less common in single-domain DGC proteins. In an analysis of DGC proteins in 867 prokaryotic genomes, about 66% of the DGC single-domain proteins had the GGEEF motif [33]. It has been shown that, in general, I sites are less common in catalytically active DGC hybrid proteins, which has led to the hypothesis that these proteins have lower activities compared to single-domain DGCs, sparing them the need for an I site [33]. Furthermore, 20% of the proteins (11 copies) were found to have degenerate GGDEF domains, two of which, were single-domain GGDEF proteins (KPK_A0039 in Kp342 and KPN_pKPN3p05901 in MGH 78578) [See Additional file 1. Other hybrid proteins with a degenerate GGDEF domain included KPK_0227 in Kp342, and its homologs in the clinical strains, that had a conserved EAL domain, and proteins KPK_1394 and KPK_0458 in Kp342, and their homologs in the other two strains, that had degenerate GGDEF and EAL domains. Some of these proteins also had additional domains like HAMP and MASE.

Several GGDEF degenerate proteins have been studied in other bacteria. They usually lack DGC activity but in many cases have adopted different functions, some of which involve binding of c-di-GMP [33]. The LapD protein in *Pseudomonas fluorescens*, for instance, has degenerate and enzimatically inactive GGDEF and EAL domains but acts as a c-di-GMP effector protein that modulates biofilm formation. The binding of c-di-GMP to its degenerate EAL domain induces conformational changes of its HAMP domain, resulting in the secretion and localization of the LapA adhesin required for attachment and biofilm formation [34]. Protein CC3396 from *C. crescentus* is a hybrid protein that harbors a degenerate GGDEF domain that is able to bind GTP and subsequently activate PDE activity in the associated EAL domain [35]. Characterization of the degenerate GGDEF proteins in *K. pneumoniae* might therefore reveal interesting novel functions in this bacterium.

### Comparative analysis of GGDEF and EAL containing genes

**Figure 2:**
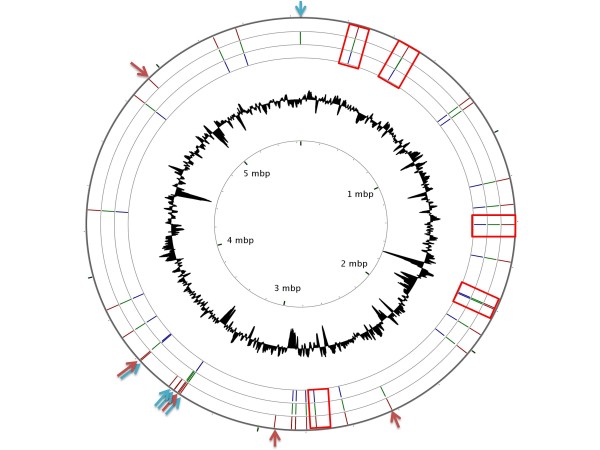
**DGCs and PDEs present in the genomes of*K. pneumoniae*342, MGH 78578 and NTUH K2044.** The distribution of GGDEF and EAL domain-containing proteins is shown. The circles represent each genome with lines indicating the DGC and PDE present: red lines for *K. pneumoniae* 342, green lines for MGH 78578 and blue lines for NTUH-K2044. The inner-most circle shows genome positions and the next to last circle shows the GC content. Arrows indicate exclusive copies or copies found in only two of the three genomes, blue arrows for PDEs and red arrows for DGCs, and rectangles represent hybrid proteins with GGDEF and EAL domains. The circular map was generated using the CGView Server [36], with the following parameters: blastx, expect = 0.00001, alignment_cutoff = 85, identity_cutoff = 85.

We next compared the GGDEF and EAL-encoding genes in the three sequenced genomes available. There were 15 genes for GGDEF proteins common to all genomes, which had more than 90%, identity at the amino acid level (Figure 2). The shared genes could be involved in diverse phenotypes important for cell growth and survival in different environments, some of which could be important for virulence properties, as has been described in other bacterial pathogens [24]. Interestingly, the gene for YfiN (KP1_4180), a protein recently found to have catalytic activity and to be implicated in pili production and biofilm formation [15], was found in all genomes. Several studies have also shown that environmental *Klebsiella* isolates can be as virulent as clinical strains [2], indicating that they harbor determinants involved in pathogenesis. Four of these GGDEF-containing proteins, one from the environmental strain Kp342 (KPK_A0039), two from strain MGH 78578 (KPN_pKPN3p05967 and KPN_pKPN3p05901) and one from strain NTUH-K2044 (pK2044_00660) were plasmid encoded [See Additional file 1. Of these, only KPK_A0039 had a homologous gene in the chromosome of Kp342, while KPN_pKPN3p05967, KPN_pKPN3p05901 and pK2044_00660 were unique genes in their respective strains. These genes could therefore have been acquired through horizontal gene transfer, a mechanism common in acquisition of drug resistance in *K. pneumoniae* clinical strains. Of the three, the gene (KPN_pKPN3p05901) had degenerate A and I sites and probably lacks catalytic activity; alternative functions, such as being a c-di-GMP effector protein, would have to be further analyzed.

In addition to shared genes for GGDEF proteins, there were three genes exclusive to the environmental strain Kp342 (KPK_3356, KPK_4891 and KPK_2890) and two additional genes in this strain (KPK_3558 and KPK_3323) that had homologs in only one of the other two genomes analyzed (Figure 2). Gene KPK_3558 had 99% identity at the amino acid level with gene KP1_1983 of *K. pneumoniae* NTUH-K2044, and KPK_3323 had 98% amino acid identity with gene KPN_01163 from *K. pneumoniae* MGH 78578. The three copies found exclusively in the environmental strain Kp342 could be important for interactions with plants and the capacity to grow as a plant endophyte. In this respect, strain MGH78578 has been reported to have a limited capacity to colonize plant roots in comparison with the environmental strain Kp342 [6]. Thus, the GGDEF containing proteins found in the environmental strain could provide it with additional regulatory and functional versatility.

Although most of the PDE proteins containing the E(A/V)L motif in *K. pneumoniae* were also common to the three genomes, there were unique genes in the environmental strain Kp342 (KPK_3392 and KPK_3355) (Figure 2) and in *K. pneumoniae* MGH 78578 (KPN_00268, KPN_pKPN3p05961, KPN_pKPN4p07065 and KPN_pKPN3p05966), the latter three genes encoded on plasmids (Figure 2) [See Additional file 2. In Kp342 one gene (KPK_A0040) was found on plasmid pKP187 and had a homolog on the chromosome, and two additional genes (KPK_3327 and KPK_2809) had homologs in only one of the other two genomes. PDE activity in *K. pneumoniae* has been demonstrated only in a few cases: MrkJ (KP1_4554) and BlrP1 (KPN_01598) [13,15]. From our analysis it therefore appears that the environmental strain Kp342 has more copies of GGDEF/EAL proteins than the clinical isolates. Future studies focused on the function of many of these DGC and PDE genes might shed light on the processes involving growth and survival of this bacterium under different environmental settings.

**Figure 3:**
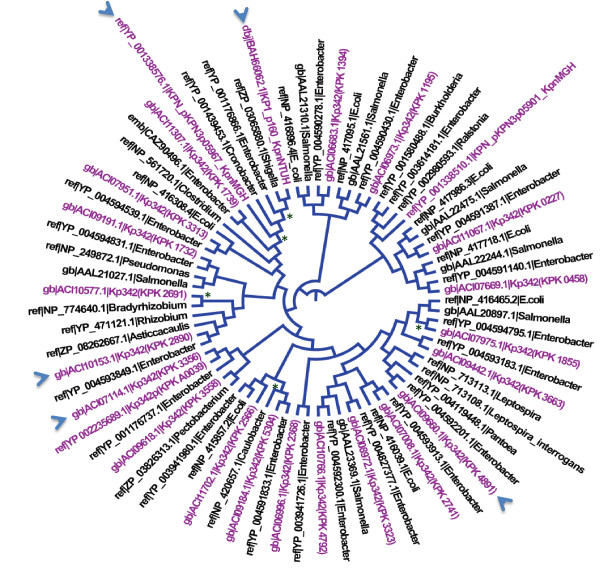
**Phylogeny of*K. pneumoniae*GGDEF proteins.** The phylogenetic reconstruction was done using neighbor-joining with 73 amino acid sequences from *K. pneumoniae* GGDEF proteins and other bacteria. Nodes with less than 70% support after 1000 bootstrap replicates are indicated with an asterisk. GGDEF proteins from Kp342, MGH78578 and NTUH-K2044 are highlighted in purple. Arrowheads represent the unique GGDEF proteins found in the *K. pneumoniae* strains 3 genomic and 3 plasmic encoded copies. The scale bar indicates the number of amino acid substitutions per site.

To further analyze the GGDEF proteins in *K. pneumoniae*, we constructed a phylogenetic tree using protein sequences from *K. pneumoniae* and other bacteria (Figure 3). This analysis showed that most of the GGDEF proteins grouped with proteins from other organisms and not with one another. However, KPK_3356, which is unique in the Kp342 genome, was closely related to KPK_A0039 and had 96% amino acid sequence identity. Interestingly, KPK_A0039 is on plasmid pKP187 of the same strain Kp342 [See Additional file 1 and could therefore have resulted from an event of horizontal gene exchange and a transfer between the plasmid and the chromosome. Other unique GGDEF proteins in Kp342, like KPK_4891 and KPK_2890, were close to GGDEF proteins from *Enterobacter sp*., with more than 96% amino acid sequence identity (Figure 3). The GGDEF proteins KPN_pKPN3p05967 and KPN_pKPN3p05901, found on plasmid pKPN3 of MGH78578, also grouped with GGDEF proteins of *Enterobacter sp.*, whereas pK2044_00660, found on plasmid pK2044 of NTUH-K2044, grouped with GGDEF proteins from *Shigella sp*. (Figure 3). These results suggest that many of these proteins are phylogenetically related, perhaps because they are derived from a common ancestor or due to horizontal gene transfer events between *K. pneumoniae* and other bacteria [37]. Additional studies would need to be carried out to further understand the diversity and distribution of GGDEF proteins in these organisms.

## Conclusions

As in other enteric bacteria, *K. pneumoniae* harbored multiple copies of GGDEF and EAL-containing proteins. Recent studies have elucidated functions associated with some of these proteins, but much remains to be known in terms of their regulation and involvement in specific cellular functions. Some of the sensor domains identified, such as MASE, CHASE, CACHE and the CSS-motif have not been well characterized to date. In contrast to other well-studied microorganisms, such as *C. crescentus* and *P. aeruginosa*, no REC domains were identified. The phylogenetic analysis also indicated similarity with GGDEF proteins from other bacteria, which raises questions regarding the origin and distribution of these copies among multiple bacterial species*.* This analysis therefore shows parallels and differences with other bacteria and the presence of multiple proteins with diverse domain architecture that is indicative of a complex c-di-GMP network in *K. pneumoniae.* Future studies focused on the function of many of these DGC and PDE proteins might shed light on the processes involving growth and survival of this bacterium in different environmental settings.

## Methods

The analysis was carried out with the following genomes: *K. pneumoniae* Kp342, *K. pneumoniae* MGH 78578 and *K. pneumoniae* NTUH-K2044 (GenBank NC_011283, NC_009648 and NC_012731, respectively). Genes coding for proteins with the GG(D/E)EF and E(A/V)L sequence motifs were identified with PSI-BLAST [38] using reference sequences available at NCBI Gene Entrez [39] [See Additional file 1, against the three *K. pneumoniae* genomes. Input sensory domains were identified using the databases CDD at the NCBI [40], InterproScan [41], pFam [42] and SMART [43]. Transmembrane segments were identified using SMART and SOSUIsignal [43,44], and the presence and localization of signal peptides was predicted using the SignalP 3.0 Server and SOSUIsignal [44,45]. Multiple alignments were done with the program MUSCLE [46] to identify the I site in each of the *K. pneumoniae* GGDEF domain proteins. Finally, the Genomic BLAST database from NCBI [38] was used to identify homologous GGDEF/EAL proteins in these three genomes. For all homologous proteins, Blastp was performed and the following parameters were considered: E-value greater than 10^-6^, identity percentage less than 85% and query coverage greater than 95%. The homologous protein obtained was validated by Random Shuffling through PRSS/PRFX, using 500 shuffles [47]. The phylogenetic reconstruction was done with MEGA 5.05 [48], using 73 amino acid sequences and the neighbor-joining method with 1000 bootstrap replicates. Sequences from other families of Bacteria were selected from the Signaling Census database [20].

The logo sequences were generated using WebLogo 3.0 [49]. For DGCs we used an alignment of 9 DGC sequences [GenBank: YP_653766.1, YP_002517919.1, YP_258266.1, NP_252391.1, YP_631414.1, YP_471572.1, NP_459380.1, NP_463410.1, NP_416465.2] and 40 *K. pneumoniae* single-domain DGCs identified here. The logo for the PDE domain was done from an alignment of 7 PDE sequences [GenBank: AAC23902.1, AAC76550.2, ABJ13888.1, AAG07334.1, ACP09769.1, AAC73418.1, CAB13282.1] and 40 *K. pneumoniae* PDEs identified here. The alignments were done using MUSCLE [46].

## Abbreviations

DGC, diguanylate cyclase; PDE, phosphodiesterase; c-di-GMP, Bis-(3’-5’)-cyclic dimeric GMP; REC, receiving domain with phosphoacceptor site; CACHE, Ca2+ channels and chemotaxis receptors domain; CHASE, cyclase/histidine kinases-associated sensing extracellular domain; MASE, Membrane-associated sensor domain; PAS, PER, ARNT and SIM domain; HAMP, Histidine kinases, Adenylyl cyclases, Methyl binding proteins, Phosphatases domain; GAF, cGMP phosphodiesterase, adenylyl cyclase domain; BLUF, Sensing blue-light using FAD.

## Competing interests

The authors declare that they have no competing interest.

## Authors’ contribution

The bioinformatics analysis was carried out by DC, analysis of results and discussions were done by DC, MH, ML, LZ and MMZ, the manuscript was prepared by DC, MH, ML, LZ and MMZ. All authors read and approved the final manuscript.

## Supplementary Material

Additional file 1 **Title: Inventory of GGDEF proteins in*K. pneumoniae*342, MGH 78578 and NTUH-K2044.** Click here for file

Additional file 2 **Title: Inventory of EAL proteins in*K. pneumoniae*342, MGH 78578 and NTUH-K2044.** Click here for file
