# Supplementary material for: Comparative analysis of diguanylate cyclase and phosphodiesterase genes in Klebsiella pneumoniae
Source: BMC Microbiol. 2012 Jul 9;12:139. doi: 10.1186/1471-2180-12-139 (PMC3431978; doi:10.1186/1471-2180-12-139)
Supplement: Additional file 1 — Title: Inventory of GGDEF proteins inK. pneumoniae342, MGH 78578 and NTUH-K2044. [file 1471-2180-12-139-S1.pdf]

Klebsiella pneumoniae 342						
**#**	**Accession number**	**Gen symbol**	**Type of protein**	**Protein size**	**Domain location**	**Gene location**
1	ACI07975.1	KPK_1855	GGDEF-only	594aa	428 – 586	Chromosome
			Cache_1		221 – 290	
2	ACI09442.1	KPK_3663	GGDEF-only	379 aa	222 – 377	Chromosome
3	YP_002235689.1	KPK_A0039	GGDEF-only	352 aa	199 – 348	plasmid pKP187
			MASE2		20 – 110	
4	ACI07114.1	KPK_3356	GGDEF-only	352 aa	199 – 348	Chromosome
			MASE2		20 – 110	
5	ACI09660.1	KPK_4891	GGDEF-only	464 aa	302 – 457	Chromosome
6	ACI09618.1	KPK_3558	GGDEF-only	306 aa	151 – 297	Chromosome
7	ACI11702.1	KPK_2566	GGDEF-only	418 aa	255 – 400	Chromosome
			PAS_4		138 – 244	
8	ACI06996.1	KPK_2368	GGDEF-only	453 aa	293 – 446	Chromosome
9	ACI07008.1	KPK_2741	GGDEF-only	474 aa	311 – 465	Chromosome
10	ACI08972.1	KPK_3323	GGDEF-only	594 aa	428 – 586	Chromosome
			HAMP		188 – 254	
			GAF		288 – 416	
11	ACI10766.1	KPK_4792	GGDEF-only	348 aa	190 – 343	Chromosome
12	ACI07951.1	KPK_3313	GGDEF-only		176 – 329	Chromosome
			GAF		19 – 164	
13	ACI11307.1	KPK_1739	GGDEF-only	323 aa	167 – 322	Chromosome
			GAF		28 – 155	
14	ACI06973.1	KPK_1195	GGDEF-only	407 aa	249 – 401	Chromosome
			HAMP		156 – 232	
15	ACI09184.1	KPK_5304	GGDEF-only	510 aa	357 – 507	Chromosome
			Cache_1		156 – 231	
16	ACI09191.1	KPK_1732	GGDEF-hybrid	636 aa	227 – 383	Chromosome
			EAL-hybrid		402 – 635	
17	ACI10577.1	KPK_2691	GGDEF-hybrid	551 aa	137 – 286	Chromosome
			EAL-hybrid		310 – 544	
			PAS		10-122	
18	ACI10153.1	KPK_2890	GGDEF-hybrid	718 aa	330 – 454	Chromosome
			EAL-hybrid		476 – 709	
			CHASE4		59 – 223	
19	ACI11067.1	KPK_0227	GGDEF-hybrid	657 aa	235 – 385	Chromosome
			EAL-hybrid		403 – 642	
			HAMP		165 – 220	
20	ACI06683.1	KPK_1394	GGDEF-hybrid	723 aa	324 – 463	Chromosome
			EAL-hybrid		490 – 718	
			MASE1		13– 314	
21	ACI07669.1	KPK_0458	GGDEF-hybrid	646 aa	227 – 382	Chromosome
			EAL-hybrid		403 – 634	

Klebsiella pneumoniae subsp. pneumoniae MGH 78578						
**#**	**Accession number**	**Gen symbol**	**Type of protein**	**Protein size**	**Domain location**	**Gene location**
1	ABR77847.1	KPN_02424	GGDEF-only	559 aa	395 – 554	Chromosome
			Cache_1		221 – 290	
2	ABR76335.1	KPN_00899	GGDEF-only	350 aa	193 – 348	Chromosome
3	ABR77225.1	KPN_01794	GGDEF-only	418 aa	255 – 400	Chromosome
			PAS_4		139 – 244	
4	ABR77069.1	KPN_01638	GGDEF-only	474 aa	311 – 465	Chromosome
5	YP_001338576.1	KPN_pKPN3p05967	GGDEF-only	318 aa	164 – 318	plasmid pKPN3
			GAF		27 – 154	
6	ABR76596.1	KPN_01163	GGDEF-only	594 aa	428 – 586	Chromosome
			GAF		287– 424	
			HAMP		188 – 254	
7	ABR77411.1	KPN_01980	GGDEF-only	391 aa	231 – 384	Chromosome
8	ABR80168.1	KPN_04822	GGDEF-only	269 aa	111 – 264	Chromosome
9	ABR77868.1	KPN_02450	GGDEF-only	323 aa	167 – 322	Chromosome
			GAF		28 – 155	
10	ABR76605.1	KPN_01172	GGDEF-only	337 aa	176 – 329	Chromosome
			GAF		19 – 164	
11	ABR79729.1	KPN_04370	GGDEF-only	510 aa	357 – 507	Chromosome
			Cache_1		156 – 231	
12	ABR78331.1	KPN_02925	GGDEF-only	407 aa	249 – 401	Chromosome
			HAMP		182 – 235	
13	YP_001338510.1	KPN_pKPN3p05901	GGDEF-only	249 aa	1 – 70	plasmid pKPN3
14	ABR77108.1	KPN_01677	GGDEF-hybrid	551 aa	137 – 286	Chromosome
			EAL-hybrid		310 – 544	
			PAS		10 – 122	
15	ABR77873.1	KPN_02455	GGDEF-hybrid	595 aa	186 – 342	Chromosome
			EAL-hybrid		361 – 594	
16	ABR79266.1	KPN_03879	GGDEF-hybrid	657 aa	235 – 385	Chromosome
			EAL-hybrid		403 – 642	
			HAMP		149 – 222	
17	ABR78161.1	KPN_02745	GGDEF-hybrid	737 aa	324 – 447	Chromosome
			EAL-hybrid		491 – 723	
			MASE1		13 – 287	
18	ABR79052.1	KPN_03660	GGDEF-hybrid	646 aa	227 – 382	Chromosome
			EAL-hybrid		403 – 634	

Klebsiella pneumoniae subsp. pneumoniae NTUH-K2044						
**#**	**Accession number**	**Gen symbol**	**Type of protein**	**Protein size**	**Domain location**	**Gene location**
1	BAH64152.1	KP1_3555	GGDEF-only	559 aa	395 – 554	Chromosome
			Cache_1		221 – 289	
2	BAH62594.1	KP1_1864	GGDEF-only	379 aa	222 – 377	Chromosome
3	BAH63494.1	KP1_2841	GGDEF-only	459 aa	296 – 441	Chromosome
			PAS_4		180 – 285	Chromosome
4	BAH63340.1	KP1_2678	GGDEF-only	474 aa	311 – 465	Chromosome
5	BAH63690.1	KP1_3053	GGDEF-only	489 aa	329 – 482	Chromosome
6	BAH62704.1	KP1_1983	GGDEF-only	306 aa	151 – 297	Chromosome
7	BAH64237.1	KP1_3652	GGDEF-only	323 aa	167 – 322	Chromosome
			GAF		28 – 155	
8	BAH61623.1	KP1_0782	GGDEF-only	348 aa	190 – 343	Chromosome
9	BAH62890.1	KP1_2191	GGDEF-only	337 aa	176 – 329	Chromosome
			GAF		19 – 164	
10	BAH61129.1	KP1_0227	GGDEF-only	510 aa	357 – 507	Chromosome
			Cache_1		156 –231	
11	BAH64717.1	KP1_4180	GGDEF-only	407 aa	249 – 401	Chromosome
			HAMP		182 – 235	
12	BAH66062.1	pK2044_00660	GGDEF-only	324 aa	164 – 318	plasmid pK2044
			GAF		28 – 155	
13	BAH63385.1	KP1_2724	GGDEF-hybrid	562 aa	148 – 297	Chromosome
			EAL-hybrid		321 – 555	
			PAS		21 – 133	
14	BAH64244.1	KP1_3659	GGDEF-hybrid	636 aa	227 – 383	Chromosome
			EAL-hybrid		402 – 635	
15	BAH65435.1	KP1_4976	GGDEF-hybrid	646 aa	227 – 382	Chromosome
			EAL-hybrid		403 – 634	
16	BAH64545.1	KP1_3998	GGDEF-hybrid	723 aa	324 – 447	Chromosome
			EAL-hybrid		491 – 723	
			MASE1		13 – 287	
17	BAH65655.1	KP1_5220	GGDEF-hybrid	657 aa	235 – 385	Chromosome
			EAL-hybrid		403 – 642	
			HAMP		149 – 222	
