# Supplementary material for: Comparative analysis of diguanylate cyclase and phosphodiesterase genes in Klebsiella pneumoniae
Source: BMC Microbiol. 2012 Jul 9;12:139. doi: 10.1186/1471-2180-12-139 (PMC3431978; doi:10.1186/1471-2180-12-139)
Supplement: Additional file 2 — Title: Inventory of EAL proteins inK. pneumoniae342, MGH 78578 and NTUH-K2044. [file 1471-2180-12-139-S2.pdf]

Klebsiella pneumoniae 342						
**#**	**Accession number**	**Gen symbol**	**Type of protein**	**Protein size**	**Domain location**	**Gene location**
1	ACI10331.1	KPK_5207	EAL-only<br>CSS-motif	534 aa	272 – 506<br>36 – 247	Chromosome
2	ACI07372.1	KPK_1552	EAL-only<br>CSS-motif	520 aa	264 – 500<br>37 – 245	Chromosome
3	ACI06700.1	KPK_1962	EAL-only<br>CSS-motif	523 aa	270 – 501<br>40 – 245	Chromosome
4	ACI11350.1	KPK_3563	EAL-only<br>CSS-motif	511 aa	254 – 490<br>33 – 229	Chromosome
5	ACI09821.1	KPK_4255	EAL-only<br>CSS-motif	519 aa	272 – 502<br>35 – 247	Chromosome
6	ACI07166.1	KPK_2789	EAL-only<br>BLUF	405 aa	157 – 386<br>2 –94	Chromosome
7	ACI08361.1	KPK_3794	EAL-only<br>BLUF	403 aa	162 – 389<br>2 – 94	Chromosome
8	ACI12139.1	KPK_A0040	EAL-only	230 aa	1 – 223	plasmid pKP187
9	ACI08928.1	KPK_3355	EAL-only	230 aa	1 – 223	Chromosome
10	ACI07129.1	KPK_1302	EAL-only<br>MASE1	742 aa	497 –730<br>15 – 321	Chromosome
11	ACI11269.1	KPK_3392	EAL-only<br>GAF	587 aa	333 – 569<br>26 – 155	Chromosome
12	ACI11146.1	KPK_0810	EAL-only	413 aa	159 – 391	Chromosome
13	ACI06911.1	KPK_0837	EAL-only	238 aa	5 – 226	Chromosome
14	ACI11208.1	KPK_3327	EAL-only	223 aa	1 – 216	Chromosome
15	ACI09771.1	KPK_2809	EAL-only<br>BLUF	405 aa	157 – 386<br>2 - 94	Chromosome

Klebsiella pneumoniae subsp. pneumoniae MGH 78578						
**#**	**Accession number**	**Gen symbol**	**Type of protein**	**Protein size**	**Domain location**	**Gene location**
1	ABR79816.1	KPN_04461	EAL-only	534 aa	272 – 506	Chromosome
			CSS-motif		36 – 247	
2	ABR80340.1	KPN_pKPN3p05961	EAL-only	484 aa	235 – 469	plasmid pKPN3
			CSS-motif		2 – 210	
3	ABR75721.1	KPN_00268	EAL-only	503 aa	241 – 473	Chromosome
			CSS-motif		12 – 216	
4	ABR78027.1	KPN_02609	EAL-only	520 aa	264 – 500	Chromosome
			CSS-motif		37 – 245	
5	ABR77757.1	KPN_02331	EAL-only	527 aa	270 – 501	Chromosome
			CSS-motif		40 – 245	
6	ABR75877.1	KPN_00425	EAL-only	519 aa	272 – 502	Chromosome
			CSS-motif		35 – 247	
7	ABR76445.1	KPN_01010	EAL-only	357 aa	124 – 357	Chromosome
8	ABR77029	KPN_01598	EAL-only	405 aa	159 – 386	Chromosome
			BLUF		2 – 94	
9	ABR80444.1	KPN_pKPN4p07065	EAL-only	235 aa	11 – 219	plasmid pKPN4
10	ABR76218.1	KPN_00782	EAL-only	403 aa	160 – 389	Chromosome
			BLUF		2 – 94	
11	ABR80345.1	KPN_pKPN3p05966	EAL-only	56 aa	1 – 41	plasmid pKPN3
12	ABR78238.1	KPN_02828	EAL-only	742 aa	497 – 730	
			MASE1		15 – 321	
13	ABR78692.1	KPN_03294	EAL-only	470 aa	216 – 448	Chromosome
14	ABR78672.1	KPN_03274	EAL-only	238 aa	5 – 226	Chromosome
15	ABR76592.1	KPN_01159	EAL-only	228 aa	1 – 222	Chromosome

Klebsiella pneumoniae subsp. pneumoniae NTUH-K2044						
**#**	**Accession number**	**Gen symbol**	**Type of protein**	**Protein size**	**Domain location**	**Gene location**
1	BAH62699.1	KP1_1978	EAL-only	511 aa	254 – 490	Chromosome
			CSS-motif		33 – 224	
2	BAH64058.1	KP1_3456	EAL-only	530 aa	273 – 504	Chromosome
			CSS-motif		43 – 248	
3	BAH64404.1	KP1_3839	EAL-only	520 aa	264 – 500	Chromosome
			CSS-motif		37 – 245	
4	BAH62079.1	KP1_1299	EAL-only	519 aa	272 – 502	Chromosome
			CSS-motif		35 – 247	
5	BAH65077.1	KP1_4578	EAL-only	413 aa	159 – 391	Chromosome
6	BAH63290.1	KP1_2624	EAL-only	422 aa	176 – 403	Chromosome
			BLUF		19 – 111	Chromosome
7	BAH61218.1	KP1_0324	EAL-only	534 aa	272 – 506	
			CSS-motif		36 – 247	
8	BAH62467.1	KP1_1729	EAL-only	403 aa	160 – 389	Chromosome
			BLUF		2 – 94	
9	BAH65056.1	KP1_4554	EAL-only	238 aa	5 – 226	Chromosome
10	BAH64624.1	KP1_4080	EAL-only	742 aa	498 – 735	Chromosome
			MASE1		15 – 321	
